# Translating epigenetics into clinic: focus on lupus

**DOI:** 10.1186/s13148-017-0378-7

**Published:** 2017-08-02

**Authors:** Zijun Wang, Christopher Chang, Mou Peng, Qianjin Lu

**Affiliations:** 1 Department of Dermatology, The Second Xiangya Hospital, Central South University, 139 Renmin Road, Changsha, Hunan 410011 China; 2 Division of Rheumatology, Allergy and Clinical Immunology, University of California, Davis, CA USA; 3 Department of Urology, The Second Xiangya Hospital, Central South University, Changsha, Hunan China

**Keywords:** Epigenetic modification, Biomarker, DNA methylation, Histone modification, MicroRNAs, Systemic lupus erythematosus

## Abstract

Systemic lupus erythematosus (SLE) is a chronic relapsing–remitting autoimmune disease with highly heterogeneous phenotypes. Biomarkers with high sensitivity and specificity are useful for early diagnosis as well as monitoring disease activity and long-term complications. Epigenetics potentially provide novel biomarkers in autoimmune diseases. These may include DNA methylation changes in relevant lupus-prone genes or histone modifications and microRNAs to upregulate and downregulate relevant gene expression. The timing and nature of epigenetic modification provide such changes. In lupus, DNA methylation alterations in cytokine genes, such as IFN-related gene and retrovirus gene, have been found to offer biomarkers for lupus diagnosis. Histone modifications such as histone methylation and acetylation lead to transcriptional alterations of several genes such as PTPN22, LRP1B, and TNFSF70. There are varieties of microRNAs applied as lupus biomarkers, including DNMT1-related microRNAs, renal function-associated microRNAs, microRNAs involved in the immune system, and microRNAs for phenotype classification. Thus, we conclude a wide range of promising roles of epigenetic biomarkers aiding in the diagnosing and monitoring of lupus diseases and the risk of organ damage.

## Background

Systemic lupus erythematosus (SLE) is a prototype of the autoimmune diseases presenting a variety of immunological features and clinical behaviors characterized by an autoantibody response to nuclear and cytoplasmic antigens. Clinical symptoms such as arthralgia or arthritis, skin lesions, and systemic disorders are typical manifestations of organ damages in the joint, kidney, central nervous system, heart, etc., [1]. Although precise mechanisms of pathogenesis and the development of specific clinical patterns are largely unknown, there still seems to follow a defined schema of progression as starting from a preclinical phase of disease to pathologic autoimmunity, developing into distinct organ dysfunction.

There is a cluster of autoantibodies involved in the pathogenesis of autoimmune diseases, where they function against nuclear antigens [2]. SLE is facilitated by generating numerous autoantibodies, particularly unusual serum antinuclear antibodies (ANAs), antibodies directed against double-stranded DNA (anti-dsDNA), anti-histone antibodies, anti-SSA/Ro and anti-SSB/La antibodies, anti-phospholipid (aPL) antibodies, or anti-Smith (anti-Sm) nuclear antigens in abnormal titer. These autoantibodies are more likely to be disease biomarkers and clinical predictors as they are associated with some clinical features and disease phenotype prediction [3–6]. Anti-dsDNA is among the most common biomarkers for the early diagnosis of SLE, given that it is frequently occurring in most patients’ serum samples, before and after the diagnosis. Also, anti-dsDNA antibody is fluctuated with, and correlates with, the kidney. A study of 40 untreated patients with lupus nephritis indicated a clear correlation between the presence of anti-dsDNA and disease severity on renal biopsy [7]. These data link the anti-dsDNA antibody levels to the Systemic Lupus Erythematosus Disease Activity Index (SLEDAI) score and lupus flare. The test of anti-dsDNA antibody is vital and remains an interesting research to observe, and besides being widely used in the clinical diagnosis, it is also considered to track the patient’s progress and investigate the pathogenesis and development of diseases [8]. Also, the fluctuation of the level of anti-dsDNA antibodies and complement proteins is tightly associated with disease activity and therapeutic effect [9–15]. Anti-histone antibodies are often found in a special type of lupus which is caused by certain medicine. Anti-SSA/Ro and anti-SSB/La antibodies have been suggested to be closely related to neonatal lupus erythematosus and photosensitivity. Anti-phospholipid antibodies are clinically associated with anti-phospholipid syndrome (APS) which is a typical autoimmune disease [16]. In addition, antibodies to Smith are highly specific for SLE. The pathogenic role and the contribution of anti-Sm antibodies are still not very clear [17]. But, its high specificity for SLE diagnosis indicates important immunological diagnostic criteria for the disease. However, the sensitivity is not very high and less than 20% of Caucasian SLE patients and about 30–40% of African, African-American, and Asian patients can be detected in a multi-ethnic lupus cohort with 2322 patients [18]. As to ANA, a phase II randomized study has pointed out that ANAs are found to be negative in more than 20% of SLE patients in a period of time during the disease process [19, 20]. On the other hand, it can be found positive within a range of autoimmune diseases, such as Sjogren’s syndrome (SS), scleroderma, and rheumatoid arthritis. In the case of this scenario, although ANA has been widely applied as a serological marker for diagnosis of SLE for many years, its value has been sometimes unreliable for its poor specificity [21, 22]. In a retrospective study, more than 90% of patients who were referred to a tertiary rheumatology clinic for a positive ANA test result had no evidence for an ANA-associated rheumatic disease. The poor predictive value of a positive ANA was largely due to unnecessary testing in patients with low pretest probabilities for an ANA-associated rheumatic disease [23]. Beyond that, there are many other types of diagnostic indicators in lupus, including acute-phase proteins, erythrocyte sedimentation rate (ESR), C-reactive protein (CRP), and complement protein level, but little is known about the stability and accuracy of them [24].

**Fig. 1:**
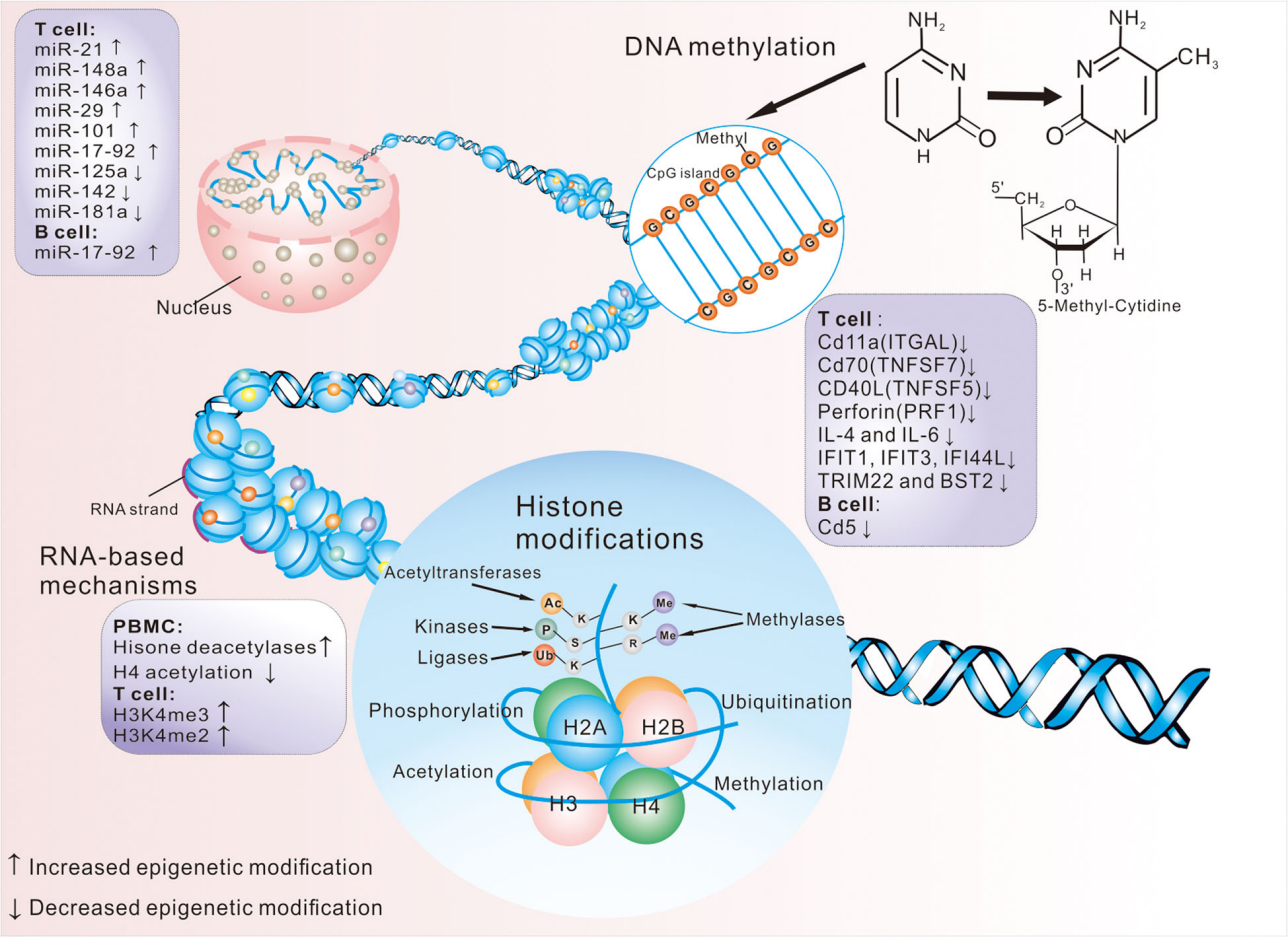
A description of the three main epigenetic mechanisms involved in SLE pathogenesis: DNA methylation, histone modification, and RNA-based mechanisms, which can alter genome and generate various gene expression profiles. DNA methylation is catalyzed by transferring a methyl group to the five positions of cytosine in DNA. Histone modifications refer to covalent posttranslational modifications of the nucleosomal histones H2A, H2B, H3, and H4, with one H3–H4 tetramer and two H2A–H2B dimers. The lysine and arginine residues of histone proteins that extrude from the nucleosome can be modified via methylation, acetylation, phosphorylation, or ubiquitylation, which can be altered with variants or chemical modifications on their histone tails. The most recent mechanism of epigenetic inheritance involves some RNAs, which may play a significant role in producing higher-order chromatin structures in nucleosomal chains. Several methylation-sensitive genes (CD11a, CD70, perforin, CD40L IFN-related genes, and CD5), histone modifications (histone deacetylation, H3k4me2, and H3K4me3), and microRNAs (miR-21, miR-126, miR-148a, miR-125a, miR-142, miR-29, miR-101, miR-17–miR-92) have been noted to illustrate their involvement in lupus pathogenesis

Epigenetic modification is defined as hereditable alterations but not coincident with alterations in the underlying DNA sequence that allow biological systems to affect transcription in response to a variety of environmental stimuli. Epigenetics is manipulated by DNA methylation, histone modification, and small noncoding RNAs. In lupus, these mechanisms exert a modulating influence on variant cell types involved in the immune system and thus play a fundamental role in programming cell identity, development, and function, as well as changes of pathogenesis [25, 26]. In particular, epigenetic disequilibrium will lead to a cascade of responses to generate an altered cell nuclear activity, aberrant transcriptome, or gene expression. Studies of epigenetic aberrations in lupus derive from a large amount of works which are quite inspiring, including analysis revealing a closely connection between changes in DNA methylation patterns and twin discordance in lupus [27, 28]. There is growing evidence of the epigenetics in their utility as biomarkers and targets in new therapies. Many of the epigenetic marks have been investigated as potential SLE biomarkers, considering their easy accessibility as well as convenient and specific methods of their measurement, as well as their essential ability to modulate local inflammatory processes (Fig. 1).

The complicated characteristics and heterogeneous hallmarks result in SLE easy misdiagnosis and missed diagnosis clinically. The complex manifestation can also explain why only a small group of patients stand to benefit from the treatment. Biomarkers are efficient methods to classify patients, evaluate the potential organ damage, and predict the risk of disease process. Hence, screening for new SLE biomarkers could help physicians and other health care professionals to monitor the disease. Epigenetics involve a basic level of gene expression regulation, making them candidate biomarkers for disease activity. In addition, the new identified biomarkers could greatly impel early diagnosis and SLE treatment management. Consequently, investigating novel distinctive biomarkers is urgent and it is thus recommended that more attention be given to epigenetic biomarkers as an appropriate disease monitor.

## Main text

### DNA methylation and SLE

Methylation of DNA involves the modification that mostly described as occurring at the fifth carbon in cytosine residues of CpG dinucleotides by adding a methyl group [29]. DNA methylation is tightly associated with repressive transcriptional activity, and the process involves three de novo DNA methyltransferases (DNMTs), including DNMT3a, DNMT3b, and DNMT1. According to the function of DNMTS in the methylation process, mammalian DNMTs can be divided into two different groups. DNMT1 recognizes established methylation marks by the remethylation of hemimethylated CpGs, thereby leading to the maintenance of the DNA methylation pattern during cell division; DNMT3a and DNMT3b produce de novo methylation by adding methyl groups into previously unmethylated CpG dinucleotides [30, 31]. In mammalian genomes, the CpG dinucleotides are clustered in CpG islands, delineated as regions where the density of CG dinucleotides exceeds 50%. In general, CpG sites are involved in regulating gene expression, as they are hypomethylated in or near the promoter regions of transcriptionally active genes, while the CpG sites in inactive genes are mostly methylated. It is widely understood that increased DNA methylation results in transcriptional silencing of target genes, and there are two mechanisms in this process. One is that methylation of cytosine bases in target genes can affect the ability of transcription factors to bind to their consensual sequences; the other is the enrichment of methyl-CpG-binding domain proteins (MBDs) to methyl groups in promoter regions, functioning as both transcriptional repressor and DNA demethylase [32, 33].

DNA methylation is the most widely studied among the mechanisms of epigenetic regulation in SLE [34]. The significant role of DNA methylation involved in patients with SLE is an intense field of research. Lupus-like symptoms, such as anti-dsDNA antibodies and immune complex glomerulonephritis, can be induced in the chimerical mice after being transferred with demethylated T cells [35]. Drug-induced lupus is characterized by clinical manifestations and immunopathological serum findings similar to those of idiopathic lupus but which is temporally related to drug exposure and resolves after withdrawal of the implicated drug [36]. Two distinct DNA demethylating drugs, procainamide and hydralazine, are known to cause drug-induced lupus. Patients who take these drugs are often detected with positive serum ANA [37, 38]. DNA methylation is significantly reduced in peripheral blood mononuclear cells (PBMCs) of patients with SLE as compared to healthy controls. Accordingly, the enzymes that mediated the methylation process such as DNMT1 and MBD2 are suggested to be upregulated in SLE. Interestingly, further study has confirmed that the expression levels of the two DNA methylation enzymes are increased in SLE [39, 40].

An aberrant DNA methylation pattern triggered T and B cell responses in SLE patients [41–43]. DNA methylation in B cells helps us to find a target B cell autoreactivity in SLE. Besides, autoreactive B cells loss their capacity to produce DNA methylation that is associated with a significant prolongation of survival. It was further confirmed by another study that treating B cells with demethylating drugs would promote B cell autoreactivity [44, 45]. DNA methylation at CpG dinucleotides restricts B cell development in SLE [46]. The expression level of B lymphocyte membrane CD5 is known to augment B cell autoreactivity. CD5 is increased in SLE, due to the DNA hypomethylation [47–49].

The relationship between globally reduced DNA methylation in lupus is further confirmed when a lupus-like syndrome is induced by treating CD4+ T cells with DNA methylation inhibitors [38, 50–53]. DNA hypomethylation of T cells from active lupus patients or lupus mice has been detected, as a result of decreased expression and activity of the enzyme DNMT1 due to deficient Ras-MAPK pathway signaling [41, 54–56]. A recent study has shown a relationship between decreased DNA methylation level and enhanced activity of a catalytic subunit of protein phosphatase 2A (PP2Ac) in lupus patients. Notably, DNA methylation is dynamically modulated by PP2Ac through the phosphorylation of MEK/ERK signaling pathway [57]. The hypomethylation level increases the expression level of several methylation-sensitive genes, thereby correlating with disease activity and autoreactivity [38, 51, 58]. Further, a loss of CpG methylation can alter antigenic characteristics and induce antigenicity that could promote immune reaction and generate autoantibodies [59].

The identification of genes that are deregulated through DNA methylation changes in CD4+ T cells has long been associated with SLE autoimmunity. Multiple lines of evidence have demonstrated that gene-specific hypomethylation, especially a few methylation-sensitive autoreactivity-linked genes, plays essential roles in the pathogenesis of SLE [60]. Among them, CD11a (ITGAL) [53], perforin (PRF1) [51], CD70 (TNFSF7) [38], and CD40LG (TNFSF5) LFA-1 [61, 62] are suggested to be increased in patients with SLE. Decreased DNA methylation within ITAGAL gene promoter generates enhanced expression of CD11a that results in autoreactive lupus T cell responses [50]. This is further confirmed by the study of treating normal T cell with DNA methylation inhibitors that can induce an increased expression level of CD11a [50, 63]. As a pore-forming cytotoxic factor encoded by the PRF1 gene, perforin is also found to be overexpressed due to the hypomethylation when exposed to DNA methylation inhibitors [64]. In active SLE patients, overexpression of perforin gene is often found in CD4+ T cell, due to the demethylated CG sites within the PRF1 promoter region. In this way, increased expression of perforin assists T cells to acquire the capacity to aim and execute autologous monocytes and then connect with disease activity [51]. Likewise, CD70 is commonly found on effector T cells and cytotoxic T lymphocyte, exhibiting a lower level of DNA methylation when regarded with DNA methylation inhibitors, thus causing the overexpression of CD70 [38, 52]. CD40LG is a type II transmembrane protein encoded on the X chromosome [53]. CD40LG demethylation within the promoter region results in overexpression of CD40LG in CD4+ T cells from women with SLE [65, 66]. X chromosome inactivation caused by DNA methylation may offer part of the explanation the female predominance in SLE [53]. Beyond these cell surface molecules, DNA hypomethylation has also been identified in the promoter region of interleukin-4 (IL-4) and IL-6 genes from SLE patients, concomitant with increased expression of the two cytokines as well as disease severity [67]. Additionally, studies have found that methylation status at certain genes in lupus CD4+ T cells can be removed by growth arrest and DNA damage-induced 45alpha (Gadd45a) [68]. Gadd45a functions like an eraser to delete the methylation marks, thus inducing the dynamic demethylation process involved in cell differentiation and stress response. Furthermore, lupus flares often occurred after exposure to UV-B irradiation, and positive correlations were found between the expression of Gadd45a and hypomethylation of CD11a/CD70. Beyond that, regulatory factor X1 (RFX1) and the nuclear factor interleukin-3-regulated protein (NFIL3, also known as E4BP4) are also reported in our previous study in human lupus CD4+ T cells. Downregulation of regulatory factor X1 (RFX1) leads to CD4+ T cell autoreactivity and is responsible for the CD11a and CD70 overexpression by altering epigenetic modifications in CD4+ T cells [69]. Similarly, overexpression of the transcription regulator E4BP4 (NFIL3) may produce a protective mechanism in CD4+ T cells through inhibiting CD40L expression, thus downregulating the autoimmune responses in SLE patients [70].

A wide range of whole genome analysis suggested that interferon-related genes are hypomethylated in CD4+ T cells of SLE patients. These genes include IFIT1, IFIT3, IFI44L, TRIM22, and BST2, which are overexpressed due to a lower methylation level in activated T cells as compared to naïve T cells [71]. Interestingly, these genes involved in type I interferon signaling pathway are easy to find in both active and quiescent stages of SLE, suggesting that CD4+ T cell-specific hypomethylation is positively correlated with disease phenotypes [72].

### Histone modifications and SLE

Histones are highly conserved protein structures that form nucleosomes in eukaryotic cells to maintain DNA strand stability as well as alter gene expression. A nucleosome is the core structure of chromatin and comprises 147 bp of DNA wrapped around a histone octamer consisting of two copies each of the core histones H2A, H2B, H3, and H4.

The histone code hypothesis facilitates alterations in nucleosome rearrangement and posttranslational modifications. Histone modifications, such as histone acetylation, methylation, ubiquitination, and phosphorylation [73], have more or less been associated with SLE. Chromatin remodeling by histone modifications displays complex forms in lupus [74, 75]. Dysregulated histone modification in lupus-prone mice is a strong evidence to support the significant role of the posttranslational regulation in lupus. Histone deacetylases (HDACs), which catalyze histone deacetylation, have already been found to increase the generation of a group of cytokine genes in lupus-prone mice. The application of HDAC inhibitors, such as trichostatin A (TSA), leads to a wide-ranged acetylation of histones and thus reduces the expression level of those inflammatory cytokines, such as IL-2, interferon (IFN)-γ, and IL-6, as well as alleviate the symptoms of lupus-prone mice [76]. Therefore, the tendency of using HDAC inhibitors to treat autoimmune responses may be based on its immunosuppressive effects [77]. A recent study demonstrated that the activity of HDAC9 is increased in MRL/lpr mice; consequently, HDAC9 deficiency in MRL/lpr mice has alleviated the autoimmune response [78].

In lupus patients’ PBMC, there is evidence that an increased expression level of histone H3 lysine 4 trimethylation (H3K4me3) and histone H4 acetylation is clinically linked to SLE disease activity [79, 80]. The permissive transcriptional activity induced by histone acetylation has been negatively related with disease activity in lupus patients at an active stage [81]. In another study, cAMP-responsive element modulator (CREM)ɑ contributes to enhance Th17 cytokine production through a direct binding to certain gene loci in the promoter region of IL17A and the downregulated activity of HDAC1 and DNMT3a [82]. CREMɑ is in charge of the silencing of IL-2 in SLE T cells by interplaying with HDAC1 and DNMT, which are recruited to the promoter regions in T lymphocytes [83, 84]. Methylation of lysine 4 of histone H3 has been frequently found at promoters of genes, so it is a gene expression enhancer [85]. The expression level of CD70 is positively correlated with dimethylated H3 lysine 4 (H3K4me2) in the promoter region of TNFSF7, which is the coding gene of CD70, and this was confirmed in SLE CD4+ T cells [86].

### MicroRNAs and SLE

MicroRNAs (miRNAs) represent a large class of 21 to 23 small noncoding RNAs that play important roles in transcription activity by binding to target messenger RNAs (mRNAs). Through the complementary sequences within 3′ UTR of a transcript, miRNAs perform their function by inhibiting translational activity of target genes and/or reduce mRNA stability [87, 88]. In mammals, more than half of the protein-coding genes are found to be encoded with thousands of miRNAs. Additionally, these small noncoding RNAs are identified as fine-tuning manipulators to monitor varieties of biological processes at posttranscriptional level [88].

The dysregulation of miRNAs in SLE is a combination of internal and external factors. miRNAs play central roles in different stages of cellular activity, as well as contribute extensively to multifaceted aspects of lupus pathogenesis. miRNA expression profile exhibits a potential diagnostic use to monitor SLE as recent findings have found that the variance of miRNA expression is an efficient indicator during the disease progression. Patients with SLE have exhibited distinctive miRNA profiles as compared with healthy controls or those with other diseases. So far, screening from approximately a thousand human miRNAs, studies have concluded that their dysfunction has been related to the development and activity of diseases [89]. In addition, the unique signature has been detected in renal biopsies of lupus nephritis patients [90]. It has been demonstrated that miRNAs combined with DNA methylation to perform its function when certain miRNA targets the methylation gene DNMT1. MicroRNA profiling of T cell in lupus disclosed dysregulated miRNAs. miR-21, miR-126, and miR-148a are three DNA methylation-associated miRNAs that aimed at binding to a certain methylation machinery in SLE, and this was accomplished by directly or indirectly targeting lupus-related gene DNA methyltransferases (DNMT1). Increased expression of miR-148a and miR-126 has been found among SLE patients, which results in DNA demethylation by directly binding to DNMT1 and thus suppressing DNMT1 transcription activity [91, 92]. On the other hand, miR-21 indirectly inhibits DNMT1 activity via interacting with RASGRP1 (an enzyme that catalyzed the Ras-MAPK signaling pathway in the upstream of DNMT1). miR-142 is another methylation-regulated microRNAs which is decreased in SLE due to the enhanced expression of CpG methylation, accompanied by a permissive modulation of H3K27me3 [93]. Furthermore, miRNA-146a has a link with the disease activity of SLE, and it is a negative regulator of the type I IFN pathway in immune and inflammatory responses [94]. miR-146a downregulation is characterized by a close relationship with the inflammatory response [95].

MicroRNA can help the clinical physicians to classify the diseases according to their different phenotypes, clinical manifestations, or disease stages. The involvement of reduced miR-125a in active lupus T cells displays a negative correlation with the serum level of an inflammatory factor RANTES, which shows an elevated tendency in patients with SLE as well as playing an essential role in lupus nephropathy [96]. miR-181a is known to be downregulated in children with SLE. It has been proved that downregulation of miR-181a results in increased expression of P300/CBP-associated factor (PCAF) that is a miR-181a target gene. Further, elevated expression of PCAF could affect Hdm2 ubiquitination, which is negatively correlated with tumor suppressor protein p53, contributing to the production of apoptosis in children with SLE [97]. The miR-29 family, including miR-29a, miR-29b, and miR-29c, has been mentioned to be significant in the development and pathogenesis of autoimmune diseases. miR-29b directly influences DNMT3A and DNMT3B activity, thus producing reduced DNA methylation. Interestingly, in lupus CD4+ T cells, it has been shown that miR-29b inhibits DNMT1 by indirectly binding to Sp1 which is a permissive regulator of DNMT1 [98]. Additionally, the differential expression of hsa-miR-371-5P, hsa-miR-423-5P, hsa-miR-638, hsa-miR-1224-3P, and hsa-miR-663 in patients with SLE would provide extra information for us to classify the disease more specifically [99].

Intriguingly, there is accumulating evidence showing that miRNAs have been associated with the disease progression in lupus animal models. miR-101 is an important regulator in lupus-prone mice via modulating T cell costimulation [100], and miR-17-92-transgenic mice with overexpressed miR-17-92 in B and T lymphocytes will recruit autoantibody and lead to SLE-like manifestations [101].

### DNA methylation as biomarkers for SLE

The dysregulated methylation-sensitive genes among SLE broad our knowledge of how the methylation alterations influence the pathogenesis of SLE. Several studies provided data to support a potential role of specific DNA methylation changes as novel biomarkers for lupus disease activity. Further, the dynamic nature of DNA methylation alterations aids them to be attracting targets as disease biomarkers (Table 1).

**Table 1:** The dynamic nature of DNA methylation alterations

DNA methylation marker	Expression level of target gene	Clinical correlation and SLE disease association	Possible mechanisms	Ref.
Cytokine methylation biomarkers				
IL10 and IL1R2 hypomethylation	↑(white blood cell)	Positively correlated with the SLEDAI score	Increased level of IL-10, decreased level of IL-1	[105]
IL6 hypomethylation	↑(PBMC)	Positively correlated with renal damage and SLE flare and negatively correlated with serum C3 and C4	Increased level of IL6	[67, 107]
IFN-related gene biomarker				
IFI44L hypomethylation	↑(PBMCs)	Positively correlated with renal damage or disease activity	Increased type I IFN response signature	[110]
Specific methylation region marker				
FOXP3 TSDR methylation	↑(PBMC)	Positively correlated with the SLEDAI score and disease activity	Decreased level of nTreg cells	[113]
Retrovirus gene methylation marker				
HERV-E and HERV-K hypomethylation	↑(T cell)	Positively correlated with the SLEDAI score and anti-U1 RNP and anti-Sm antibodies, complementary, lymphopenia	Increased level of HERVs	[114, 115]

### Cytokine methylation biomarkers

IL-1 is a pro-inflammatory cytokine that plays a central role in SLE development and induction [102]. IL-1 performs its function with the help of two regulators, IL-1 receptor antagonist (IL-1RA) and type 2 IL-1 receptor (IL-1R2). IL-1R2 is a suppressor in the IL-1 signaling pathway; thus, decreased DNA methylation in the promoter region of IL-1R2 will lead to downregulation of IL-1 in SLE patients. The immune-regulatory cytokine IL-10 plays a central role in inhibiting T cell function and inflammatory response, thus forming a potential biomarker for SLE disease severity evaluation [103]. The expression level of IL-10 is elevated in the serum and tissues of SLE patients and contributes to autoantibody production and tissue damage [104].

Global methylation profiling has analyzed DNA methylation changes in white blood cells between active SLE patients and healthy donors. Among those genes, IL10 and IL1R2 genes present a range of methylation-regulated domain variance in lupus. IL10 and IL1R2 are marked with decreased methylation level in SLE as compared to the healthy controls. In addition, there is a positive correlation between decreased methylation level of IL10 and IL1R2 and greater disease activity. IL10 and IL1R2 hypomethylation could provide potential methylation indicators in the clinical diagnosis of SLE [105].

IL-6 is secreted by T cells, B cells, and macrophages, featured as a proinflammatory cytokine in immune responses. It is widely accepted that IL-6 is important in the development of inflammatory process, while dysregulation of IL-6 may lead to a pathogenic state in many autoimmune reactions [106]. Deregulated IL-6 methylation level may contribute a part to the aberrant IL-6 gene expression. DNA methylation levels of CpG locus within an IL-6 gene promoter are negatively associated with the IL-6 expression in SLE patients [67]. Recently, studies have revealed that there is a link between PBMC IL-6 expression level and lupus disease activity as compared to the IL-6 expression in PBMCs from SLE patients and healthy control. In addition, the importance of IL-6 expression in PBMCs and IL-6 methylation status was determined as it is applied both in clinical examination and experimental study. Intriguingly, decreased IL-6 hypomethylation has been deemed as an active regulator in lupus patients with kidney damage. On the other hand, hypomethylated IL-6 as well as IL-6 overexpression have been found to be negatively associated with the serum level of complements, while positively correlated with several autoantibodies and immune complexes. Thus, due to the reflection of PBMC IL-6 hypomethylation pattern in SLE, it is estimated to be a novel biological marker to predict lupus flares [107].

### IFN-related gene biomarker

IFI44L is a specific IFN-regulated gene in SLE. IFNs activate intracellular antimicrobial programs and influence the development of innate and adaptive immune responses [108]. An increased expression of type 1 IFN-regulated genes, termed IFN signature, has been well documented in the peripheral blood and tissues of SLE patients. Previous studies have shown an increase in type 1 IFN levels in SLE patients through repeated confirmation [109]. After screening for differentially methylated CpGs in a large amount of DNA samples of SLE, healthy controls, and other autoimmune diseases, studies validated significant hypomethylation of two CpG sites within the IFI44L promoter in SLE compared to healthy controls and other autoimmune diseases. In addition, the methylation levels of the two sites were significantly reduced in SLE patients with renal involvement as compared with non-renal involvement DNA methylation levels of IFI44L in the peripheral blood that may be useful in the evaluation and diagnosis of SLE. The specificity and sensitivity of IFI44L promoter methylation in discriminating between SLE and healthy controls were superior to those available tests. Moreover, IFI44L promoter methylation levels can discriminate between SLE and other autoimmune diseases such as rheumatoid arthritis and primary Sjogren’s syndrome [110].

### Specific methylation region marker

Treg cells are associated with peripheral tolerance and homeostasis in immunity. Treg cells were mentioned by Sakaguchi et al. [111] in 1995 by expressing FOXP3 and producing IL-10 and TGF-β. The suppressive function of Treg cells is mainly due to the master regulator—FOXP3. FOXP3 is the key transcriptional factor of Treg cells, without which Treg cells may lose its immune suppression function and Treg cell characteristics when developing and differentiating from naïve T cell [112]. Treg-specific demethylated region (TSDR) is an evolutionarily conserved element within the Foxp3 locus that displays completely demethylation status in natural Treg cells but hypermethylated in effector T cells.

Identifying FOXP3 TSDR methylation alterations provides us an efficient method in diagnosing and supervising the diseases activity. TSDR methylation analysis has been recognized as an available approach to monitor and quantify Treg cells in the peripheral blood and tissue. It is obvious TSDR methylation within FOXP3 promoter in active SLE patients was significantly higher than that in other diseases. As a result, it is a more effective and specific indicator in evaluating disease activity. Also, decreased TSDR methylation level combined with an enhanced number of Treg cells when patients transformed from the active stage to quiescent stage under clinical treatment. This is achieving since the physicians could directly observe the TSDR methylation level to identify disease stage. Thus, analyzing TSDR methylation status within the FOXP3 gene will be useful as a novel epigenetic index to evaluate the number and function of Treg cells and to verify different disease states [113].

### Retrovirus gene methylation marker

The human genome comprises variable long terminal repeat (LTR) elements; for example, human endogenous retroviruses (HERVs) are part of them. Amazingly, HERVs are responsible for almost approximately 8–9% of the genomic DNA. Previous works have shown an enhanced expression level of HERV-E and HERV-K gag genes in PBMCs from SLE patients [114, 115]. The expression of HERV-E clones 4–1 might be associated with the production of anti-U1 RNP and anti-Sm antibodies in patients with SLE. Furthermore, hypomethylation of HERV-E in the flanking promoter region of the CD5 gene in lupus has been found to increase CD5-E1B isotype in lupus B cells [48].

Nakkuntod et al. [116] have found that a methylation level of HERV-E LTR2C as well as HERV-K LTR5_Hs in T cells from active SLE patients was extremely lower than that in disease quiescent stage. Moreover, a decreased methylation level of HERV-E LTR2C in CD3+ CD4+ T cells has been found as a positive regulator leading to lymphopenia in active SLE. In addition, the low methylation status of HERV-K LTR5_Hs has been mentioned to be clinically related to complement activity and lupus activity. Altogether, HERV-E and HERV-K hypomethylation may be used as a prognostic marker in differentiating disease stage from active status to resting stage.

### Histone modification as biomarkers for SLE

There are various types of dysregulated histone modification marks that have been mentioned in lupus pathogenesis. However, less is known about their utility in clinical diagnosis and management. Here, we list two major markers that have been studied most widely, exhibiting an early promise as biomarkers for lupus susceptibility and monitoring (Table 2).

**Table 2:** Major markers for lupus susceptibility and monitoring

Histone modification marker	Expression level of target gene	Clinical correlation and SLE disease association	Possible mechanisms	Ref.
Histone methylation marker				
H3K4 trimethylation	↑(PBMCs)	Positively correlated with disease severity	Several candidate genes related to SLE pathogenesis (such as PTPN22, LRP1B) are involved	[79]
Histone acetylation marker				
H3K4 hypoacetylation	↑(CD4+ T cell)	Positively correlated with disease severity		[117]

### Histone methylation biomarker

Histone H3 lysine 4 trimethylation has known to be essential for studying the donation of histone modifications in regulating gene expression. H3K4me3 is commonly involved in active transcription of adjacent genes. In lupus, a genome-wide study of DNA methylation has been investigated to appear. Yet to date, information of histone lysine methylation in SLE is still insufficient.

Dai et al. [79, 90] demonstrated an important change of H3K4me3 in several crucial related candidate genes (such as PTPN22, LRP1B), which are linked to immune responses, cell signal transduction, transcriptional activity and cell apoptosis, genetic processing, and extracellular matrix, in PBMCs of SLE patients. This study also provides us with new insights into the associations between pivotal genes and histone methylation in the pathogenesis of SLE. The results indicate that H3K4me3 alterations were confirmed to play a significant role in SLE and thus could be applied as a potent clinical and biological marker as well as a hopeful target for epigenetic-based lupus treatment. Further investigations are needed to clarify the roles of identified H3K4me3 candidate genes in the pathogenesis of SLE [79].

### Histone acetylation biomarker

As mentioned above, overexpression of CD70 in SLE CD4+ T cell is mainly due to the hypomethylation of TNFSF7 promoter, which leads to the production of several autoantibodies. Studies have investigated the significant role of histone modifications in regulating CD70 expression through affecting the posttranslational marks within the TNFSF7 promoter region in lupus CD4+ T cells. In addition to DNA methyltransferase inhibitor (5-azaC), a histone deacetylase inhibitor (TSA) contributes equally to CD70 overexpression. Thus, it is not surprising that permissive histone modifications such as H3K4 hypoacetylation and H3K4 dimethylation have been found significantly increased in lupus patients. Further, the expression level of these active transcriptional regulations is positively correlated with lupus disease activity. These results state explicitly that dysregulated histone marks within the TNFSF7 promoter contribute partly to lupus pathogenesis by promoting CD70 expression, thus connecting the expression level of aberrant histone marks in T cells with disease activity [117].

### MicroRNAs as biomarkers for SLE

The close involvement of miRNAs in immunity and autoimmunity comprises a huge potential for disease pathology. Several studies showed a particularly important role of aberrant miRNAs in SLE evolution, especially in lupus nephritis; thereby, miRNA biomarkers are helpful to manage disorder and therapeutic effect.

There are a number of advantages to use miRNAs as disease indicators. The expression levels of miRNAs in serum are stable, reproducible, and consistent. In addition, compared with protein biomarkers, detection of miRNAs seems to be more available with low complexity. Evidence shows that serum miRNAs leave traces for lupus. For instance, circulating miRNAs is a significant marker to set lupus apart from other autoimmune disorders. Also, aberrant expression of miRNAs in body fluids provides us more convenience to directly determine the quantity and function of microRNAs, as a minor change of the noncoding RNA may present different clinical manifestations sometimes. Thus, testing serum or urine microRNA sequence would offer an extra efficient tool to connect small biomarkers with disease activity. This is helpful as well to investigate lupus flares and enhance disease decision-making ability in the development of SLE. Although being differentially expressed in SLE, whether miRNAs could be served as biomarkers in different clinical types of SLE still remains largely unknown (Table 3).

**Table 3:** miRNAs as biomarkers in different clinical types of SLE

MicroRNA marker	Expression level of target gene	Clinical correlation and SLE disease association	Possible mechanisms	Ref.
DNMT1-related microRNAs as a biomarker				
miR-126		Positively correlated with disease activity	Induces DNA hypomethylation	[92, 118]
miR-21		Positively correlated with the SLEDAI score, SLE flares, and remission		
miR-148a	↑(PBMCs)	Positively correlated with the SLEDAI score	Induces DNA hypomethylation	[91, 118, 119]
MicroRNA biomarkers to evaluate renal dysfunction				
miR-130b-3p	↑(serum)	Positively correlated with renal damage	Promote EMT by targeting ERBB2IP	[121]
miR-26a and miR-30b	↓(kidney and urine)	Positively correlated with disease activity	Control of mesangial cell proliferation and cell cycle-related genes	[122]
			Downregulate the anti-fibrotic protein suppressor of cytokine signaling 1 (SOCS1) and upregulate profibrotic proteins in both proximal tubular and mesangial cells	
miR-150	↑(kidney)	Positive correlation with chronicity scores		[123]
Extracellular vesicle miRNAs				
miR-26a	↑(urine exosomes) ↓(glomerular)	Positive correlation with lupus nephritis, urinary protein levels	Decreased the expression of genes associated with the podocyte differentiation and formation of the cytoskeleton	[125]
miR-29c	↓(urinary exosomes)	Negatively correlated with the histological chronicity index and glomerular sclerosis	Exacerbate renal fibrosis by targeting epithelial-to-mesenchymal transition and increasing the deposition of extracellular matrix	[126]
Immune-related microRNAs as biomarkers				
miR-146a	↓(CD4+ T cells, serum) ↑(urine)	Negative correlated with disease activity, proteinuria, lupus nephritis, GFR, histological activity index	Negative regulator in the IFN pathway	[94, 99, 132]
	Controversial (CD4+ T cells, serum) ↑(urine)	Positively correlated with proteinuria and SLEDAI score	Aim at SHIP11 to maintain an activation threshold that allows B cells to respond to antigens	[99, 118, 130, 131]
miR-155	↓(Lymphocytes) ↑miR-142-3p (plasma) ↑miR-142-5p (renal tissue)	No correlation with disease activity	Promoting T cell activity and antibody generation	
hsa-miR-142		Negatively correlated with the SLEDAI score, lupus nephritis (GFR and creatinine ratio)	Increased level of inflammatory chemokine regulated on activation, normal T cells expressed and secreted (RANTES) in SLE T cells	[90, 93, 133–135]
miR-125a	↓(CD4+ T cells, urine)			[118, 137]
miR-31	↓(T cell)	Negatively associated with diseases activity and urine protein	Reduced expression of IL-2	[139, 140]
miR-21	↑(T cell)	Positively associated with diseases activity and urine protein	T cell activation	[139]
MicroRNA biomarkers to classify disease phenotype				
hsa-miR-30e-5p, hsa-miR-92a-3p, and hsa-miR-223-3p	↑(plasma)	hsa-miR-223-3p is connected to oral ulcer and lupus anticoagulant Positively associated with serous cavity effusion. CRP and anti-Clq antibody	Not mentioned	[141]
miR-326	↑(Treg)		Regulating immune cell function	[142]

### DNMT1-related microRNAs as biomarkers

miR-126, miR-21, and miR-148a appear to be interplayed with DNMT1 and were found to be decreased in SLE CD4+ T cells as mentioned above. miR-126 is expressed only in human endothelial cells and functions to control angiogenesis. Studies have found it was markedly upregulated in PBMCs of SLE patients. It has been proved that an increased level of miR-126 in normal CD4+ T cells would lead to a decreased DNMT1 activity, as well as reduced DNA methylation level of CD11a and CD70 that contribute to their overexpression. These mechanisms are the same in lupus CD4+ T cells [92]. In lupus, miR-126 is significantly higher than healthy controls and the expression level is conversely linked to DNMT1 activity [92]. In addition, further studies have enlarged the samples and clinical trials and suggested that miR-126 is a potential biomarker [118]. Aforementioned miR-21 and miR-148a are increased in lupus CD4+ T cells with downregulated DNMT1 protein activity and decreased DNA methylation. Studies have reported that elevated expression of miR-21 in normal T cells leads to an activated disease state by generating abnormal immune responses. Accordingly, microRNA-21 silencing in vivo will ameliorate autoimmune splenomegaly in lupus mice [119]. miR-21 is significantly increased in the blood of lupus patients [91, 118], and its expression has been related to SLEDAI score [91]. Another study has also confirmed this; moreover, a longitudinal analysis of two patients showed a dramatically decreased level of miR-21 when SLE is alleviated [120]. However, the elevated degree of miR-21 is not only observed in lupus but in other autoimmune diseases, such as RA [118]. Similar to miR-21, Pan et al. demonstrated that miR-148a is significantly upregulated in human lupus CD4+ T cells as compared with healthy controls [91]. Interestingly, steroid treatment does not affect miR-148a expression in lupus T cells, which suggests that miR-148a is not sensitive to steroids or there is no connection between miR-148a expression and disease activity.

These DNA methylation-related miRNAs are part of the complex mechanisms that explain the pathogenesis of SLE with influencing different processes. The clinical use of miR-126, miR-21, and miR-148a can be considered as attractive and new miRNA biomarkers and novel therapeutic targets specific for SLE.

### MicroRNA biomarkers to evaluate renal dysfunction

The miRNA expression profiling of serum samples from early- and late-stage lupus nephritis (LN) patients as well as healthy controls was determined by microarray to measure the expression level of miR-130b-3p [121]. It is suggested that miR-130b-3p in the serum of patients with early-stage LN was remarkably increased as compared with healthy controls. Moreover, serum miR-130b-3p has been noted as a positive regulator in measuring 24-h proteinuria and renal chronicity index of early-stage LN. The application of miR-130b-3p as a marker to determine renal function and the severity of tissue damage accelerate our understanding of microRNAs in clinical use. Additionally, measuring miR-130b-3p level is also a diagnostic method to classify early and late stages of nephritis. However, there is a limitation of the connection between serum miR-130b-3p and other disease activity parameters, suggesting that miR-130b-3p may not be influenced by lupus disease activity but early renal damage only.

miR-26a and miR-30b have been reported to play a significant role in lupus nephritis. A recent study of miRNAs in lupus has identified that the expression level of miR-26a and miR-30b was downregulated in renal tissues and urine of LN patients. The downregulation of miR-26a and miR-30b is mediated by a human epidermal growth factor receptor 2 (HER-2) pathway, which is upregulated in LN and correlated with disease activity. This pathway inhibits miR-26a and miR-30b expression, thus promoting cell multiplication via suppressing cell cycle genes. miR-26a and miR-30b combined with HER-2 are considered as potential LN biomarkers, and blocking HER-2 pathway may be a promising strategy to decrease cell proliferation and damage in SLE [122].

miR-150 is a positive regulator in maturation progresses. Recent studies have shown that enforced expression of miR-150 dramatically downregulate anti-fibrotic protein suppressor of cytokine signaling 1 (SOCS1) and upregulate profibrotic proteins in both proximal tubular and mesangial cells. These findings implied that miR-150 accelerates renal fibrosis through promoting the production of profibrotic molecules induced by downregulated SOCS1. After comparing miRNA expression in kidney biopsies from patients with lupus nephritis, Zhou et al. have found there was a positive correlation between elevated miR-150 expression in renal tissues of lupus nephritis patients and high chronicity index of kidney as well as chronicity scores. Thus, miR-150 may be a promising quantitative renal biomarker for kidney injury in lupus nephritis [123].

Extracellular vesicles (EVs) are small secreted and membrane-bound subcellular compartments, typically measuring less than 1 μm in diameter. It was found that a variety of and sufficient amount of miRNAs were packaged in EVs and stably existed in almost all kinds of body fluids. EV-associated miRNA has been researched by a wide range of studies since it is a promising biomarker in renal damage in lupus patients [124]. Recent studies have found that glomerular miR-26a levels were significantly lower in patients with lupus nephritis than in healthy controls. Further, the increased expression of miR-26a in urinary exosomes is a hint of the occurrence of urine protein in patients with lupus nephritis [125]. Similarly, miR-29c levels in urinary exosomes showed a negatively correlation with the histological chronicity index and glomerular sclerosis. Moreover, miR-29c expression levels could predict the kidney damage and the degree of renal fibrosis in patients with LN [126]. Overall, miR-29c and miR-26a in exosomes correlated with the disease development of patients with LN, suggesting their convenience as predictive biomarkers of early progression in patients with LN.

### Immune-related microRNAs as biomarkers

miR-146a and miR-155 are two key regulators that aim at a group of genes involved in immune response. miR-146a is a suppressive mediator functioning in determining inflammatory responses along with miR-155 [127]. miR-155 has been mentioned to be involved in T cell differentiation, IFN-γ generation, T cell polarization especially in Th2, and antibody formation [128, 129].

Underexpression of miR-146a contributes to alterations in the type I IFN pathway in lupus patients by targeting the key signaling proteins [94]. miR-146a has been found to be downregulated in CD4+ T cells from lupus patients as compared to healthy controls, and it was conversely linked to disease prognosis. Decreased expression of miR-146a is linked to the abnormal activation of type I IFN pathway in SLE patients [94]. Recently, serum miR-146a level has been reported to decrease but increase in urine from SLE patients [99]. In the case of miR-155, evidence turns out to be quite controversial. Some studies have shown that the expression level of miR-155 is markedly increased in B and T cells [99, 130]. However, some studies showed that the expression level of miR-155 is significantly decreased in SLE patients [118, 131].

More recently, the levels of miR-146a and miR-155 in the urine sediment of SLE patients who were receiving calcitriol treatment and healthy controls were determined. The results proved that urinary miR-146a and miR-155 can be considered as a useful marker in SLE patients since their expression level is specifically higher than healthy controls. After receiving calcitriol treatment, urinary miR-155 has been found to decrease in lupus patients. In addition, the expression level of urinary miR-146a was greatly associated with estimated glomerular filtration rate, while urinary miR-155 was largely associated with proteinuria and disease activity. These findings implied that miR-146a and miR-155 have a tight association in the pathogenesis and development of SLE. Moreover, the combination of urinary miR-146a and miR-155 is a useful biomarker in evaluating disease diagnosing, disease activity, and therapeutic response [132].

hsa-miR-142, a human T cell-specific miRNA, serves as a negative modulator in T cell development as well as a suppressor in tumor progression [133, 134]. There are two transcripts produced by the miR-142: miR-142-5p is from the 5′ arm of the locus, while miR-142-3p is expressed from the 3′ arm [135]. The two miRNAs have been demonstrated to be significantly downregulated in lymphocytes from lupus patients. Nevertheless, the expression level of miR-142 showed there was no correlation with the SLEDAI score indeed [93]. Another study has reported that there is an increased expression level of circulating miR-142-3p in the plasma from SLE patients compared to healthy controls. It is suggested that elevated cellular release of miR-142-3p leads to increased miR-142-3p expression in SLE plasma through enhanced exocytosis and/or normal exocytosis of cells containing increased miRNA. Similarly, the expression level of miR-142-5p is also increased in renal biopsies of patients with lupus nephritis [90]. Basically, the mechanisms of miR-142-3p/5p in CD4+ T cells are presented as directly suppressing IL-10, CD84, as well as signaling lymphocytic activation molecule (SLAM) and SLAM-associated protein (SAP). Thus, downregulated miR-142-3p/5p in SLE CD4+ T cells restored those inflammatory cytokines as well as signaling activator and promoted T cell activity and antibody generation. Furthermore, the results were confirmed when a loss of miR-142-3p/5p in normal CD4+ T cells led to a SLE-like phenotype [93]. Interestingly, miR-142-3p has been found to be increased in monocyte-derived DCs and it is positively correlated with the generation of a range of SLE-related cytokines in lupus pathogenesis [136].

The expression level of miR-125a is significantly reduced in SLE CD4+ T cells as compared to controls. MicroRNA-125a negatively regulates RANTES expression by targeting Kruppel-like factor 13 (KLF13) in activated T cells. The reduced level of miR-125a contributes to the increased production of the inflammatory chemokines RANTES in activated T cells [96]. Similarly, circulating miR-125a-3p is decreased in SLE patients [118]. In addition, miR-125a is differentially expressed in the urine supernatant of LN children as a significantly increased expression level of miR-125a has been found in active lupus nephritis as compared with patients in non-active stage. Besides, miR-125a is mildly correlated with classical nephritis measurements such as glomerular filtration rate (GFR) and creatinine ratio. Conversely, a recent study has proved that miR-125a is upregulated in the serum sample of lupus patients, which is positively associated with the production of several inflammatory cytokines [137]. These results concluded that urinary miR-125 is a relevant and accurate biomarker of disease activity and also provides potential strategies for therapeutic intervention [138].

More recently, there is an interesting study which targeted activated T cells from Egyptian lupus patients. The study has found a decreased expression of miR-31, which was inversely associated with diseases activity and urine protein, and an increased expression level of miR-21, which is positively linked to those disease-related parameters in SLE patients. The increased expression of miR-21 in normal T cells has been noted to be related with T cell activation phenotype [139]. Moreover, the decreased expression level of miR-31 may contribute to the downregulation of IL-2, which is known to reverse the clinical manifestations of SLE [140]. The regulatory biomarkers of miR-31 and miR-21 may as well be noted as clinical biomarkers in lupus diagnosis, especially in lupus nephritis management.

### MicroRNA biomarkers to classify disease phenotype

If specifically dysregulated microRNAs in the human body could have a correspondence clinical phenotype in SLE, it would be encouraging for both the patients and the doctors and indeed achieving the goal of quick diagnosing as well as rational treating.

Recently, hsa-miR-30e-5p, hsa-miR-92a-3p, and hsa-miR-223-3p were found to increase in the plasma of SLE patients. Excitingly, there was a positive correlation between the enhanced expression of hsa-miR-223-3p and the oral ulcer as well as lupus anticoagulant [141]. Thus, it is a good way for the clinical screening and classifying patients. Another study provides a clue of how to recognize new-onset patients by measuring the expression level of miR-326, which is a regulator of immune cells and is involved in autoimmune pathogenesis and has been found significantly higher in Treg cells from SLE patients. In addition, miR-326 expression was also proved to be positively related to serous cavity effusion, CRP, and anti-C1q antibody from new-onset SLE patients compared to the new-onset patients without those syndromes [142].

## Conclusions

Epigenetics offer potentially benefits of diagnosing and management of several autoimmune diseases, such as lupus. Multiple factors in DNA methylation, histone modification, and microRNAs have already been announced with various clinical factors and phenotypes. In spite of this, epigenetic biomarkers are very convenient and operative. For example, methylation-induced change can somehow predict lupus flare. On the other hand, the medical therapy for SLE relies largely on the severity of the disease. Thus, identifying credible biomarkers for SLE will help to evaluate disease activity, verify patients at risk for organ damage, and facilitate early diagnosis and intervention to improve favorable outcomes. Furthermore, the unavoidability of aiming at lupus epigenome to offer a stable diagnostic marker is attracting, and particularly after partially revealing different crosstalk mechanisms between DNA methylation, histone modification, and miRNAs, which might be reliable epigenetic biomarkers in lupus.

## Abbreviations

DNMT: DNA methyltransferase
Gadd45a: growth arrest and DNA damage-induced 45alpha
HDAC: histone deacetylase
LN: lupus nephritis
MBDs: methyl-CpG-binding domain proteins
NFIL3: nuclear factor interleukin-3-regulated protein
PBMCs: peripheral blood mononuclear cells
RFX1: regulatory factor X1
SLE: systemic lupus erythematosus
